# Fatigue Performance and Failure Loads of Composition‐Gradient Multilayer Zirconia: Partial‐Coverage Restorations Versus Single Crowns at Different Thicknesses

**DOI:** 10.1111/jerd.70066

**Published:** 2025-12-03

**Authors:** F. A. Spitznagel, A. Marksteiner, L. S. Prott, Y. Zhang, M. B. Blatz, L. M. M. Alves, T. M. B. Campos, P. C. Gierthmuehlen

**Affiliations:** ^1^ Department of Prosthodontics Medical Faculty and University Hospital Düsseldorf, Heinrich‐Heine‐University Düsseldorf Düsseldorf Germany; ^2^ Department of Preventive and Restorative Sciences, School of Dental Medicine University of Pennsylvania Philadelphia Pennsylvania USA; ^3^ Department of Prosthodontics and Periodontology, Bauru School of Dentistry University of Sao Paulo Bauru Brazil

**Keywords:** ceramic thickness, ceramics, computer‐aided design, fatigue, partial‐coverage restoration

## Abstract

**Objective:**

This study investigated the influence of restoration type (partial‐coverage versus crown) and ceramic‐layer thickness on the fatigue behavior and failure load of monolithic composition‐gradient multilayer zirconia (4Y‐PSZ/5Y‐PSZ) molar restorations.

**Material and Methods:**

Seventy‐two CAD/CAM‐fabricated monolithic zirconia restorations (IPS e.max ZirCAD‐Prime‐Esthetic, Ivoclar‐Vivadent) were assigned to six groups (*n* = 12), based on restoration type—partial‐coverage restoration (PCR) or crown (C)—and ceramic thickness (occlusal/buccal: 0.5/0.4, 1.0/0.6, or 1.5/0.8 mm). All restorations were adhesively bonded to standardized dentin‐analogue dies. Specimens underwent thermomechanical fatigue (1.2 million cycles, 49 N, 1.6 Hz, 5°C–55°C), followed by single load to failure testing. Data were analyzed using ANOVA, Tukey's post hoc test, and independent t‐tests (α = 0.05).

**Results:**

All specimens survived fatigue. Evident cracks were observed post‐fatigue; one each in PCR‐0.5 and C‐0.5 groups, resulting in an overall success rate of 97.22%. Mean failure loads (N) were: PCR‐0.5: 2047, PCR‐1.0: 2018, PCR‐1.5: 2777, C‐0.5: 695, C‐1.0: 1957, C‐1.5: 3503. Ultrathin (0.5 mm) PCRs demonstrated significantly higher failure loads than ultrathin crowns (*p* < 0.001), whereas crowns at 1.5 mm thickness outperformed PCRs (*p* = 0.005). No significant difference was observed at 1.0 mm thickness (*p* = 0.634).

**Conclusion:**

At ultrathin thicknesses (0.5 mm), partial‐coverage designs showed superior failure loads over crowns. Both restoration types are mechanically viable at 1.0 mm and 1.5 mm thicknesses.

**Clinical Significance:**

Composition‐gradient multilayer‐zirconia demonstrates high fatigue resistance and is well‐suited for minimally invasive, non‐retentive PCR molar restorations. However, a minimum thickness of 1.0 mm should be maintained for single crowns.

## 1 Introduction

Minimally invasive restorative approaches are gaining increasing importance in contemporary fixed prosthodontics, particularly for preserving sound tooth structure while maintaining mechanical integrity [1].

Partial‐coverage restorations (PCRs) have emerged as a conservative alternative to full‐coverage crowns, particularly in posterior regions subjected to high biomechanical stresses and loads [2, 3]. The clinical success of these restorations, however, is influenced by multiple factors, including material properties, restoration design, and thickness [2, 4, 5].

Occlusal veneers with non‐retentive preparations provide a minimally invasive solution for restoring occlusal surfaces compromised by bio‐corrosive defects [6]. When such defects extend to buccal and cervical regions, conventional treatment approaches such as full‐coverage crowns or occlusal restorations combined with Class V fillings are often used; however, they involve extensive tooth reduction and may exhibit compromised long‐term performance [7, 8, 9]. In such cases, extended PCRs covering occlusal, buccal, and proximal surfaces—also referred to as “full veneers”—offer a tissue‐preserving alternative that supports both functional and esthetic rehabilitation in a single restoration [10, 11].

Lithium disilicate glass–ceramics (LDS) are widely regarded as a material of choice for minimally invasive restorations, primarily due to their favorable adhesive bonding properties and clinical long‐term evidence [12, 13].

However, recent advances in zirconia ceramics offer esthetic alternatives with improved durability [14, 15, 16, 17, 18, 19]. Increasing the yttria content—from 3 mol% yttria‐stabilized tetragonal zirconia polycrystals (3Y‐TZP) to 4 mol% and 5 mol% partially stabilized zirconia (4Y‐PSZ and 5Y‐PSZ)–along with a higher cubic phase and reduced alumina content, has enhanced translucency and optical appearance [20, 21, 22, 23, 24].

This improvement in esthetics, however, comes at the cost of reduced mechanical strength and fracture toughness [25, 26, 27, 28, 29, 30, 31]. To address this trade‐off and balance the positive mechanical and optical properties of different zirconia compositions, multilayer zirconia disks with strength and translucency gradient technology have been developed to mimic the natural tooth structure [32, 33, 34, 35]. These typically combine a highly translucent 5Y‐PSZ incisal/occlusal layer with a high‐strength 3Y‐TZP or 4Y‐PSZ cervical layer to withstand functional loads [14, 36, 37, 38, 39].

Consequently, yttria‐gradient multilayer zirconia ceramics offer a versatile and esthetic solution suitable for a wide range of indications—from single crowns to multi‐unit bridges—making them a viable alternative to LDS ceramics in minimally invasive restorations. Their high strength renders them particularly advantageous in posterior regions with increased masticatory load or parafunctional activity [40, 41].

In vitro studies evaluating minimally invasive occlusal onlays with non‐retentive designs made from materials such as LDS, 3Y‐TZP, 4Y‐PSZ, and 5Y‐PSZ zirconia have demonstrated that minimal ceramic thicknesses of 0.3–0.6 mm are feasible [42, 43, 44]. Currently, only limited data are available on occlusal veneers made from gradient multilayer zirconia [43, 45]. However, so far, no studies have investigated the minimally invasive layer thicknesses required for composition‐gradient multilayer zirconia in partial‐coverage restorations involving both occlusal and buccal surfaces.

This evidence gap underscores the need for further investigation. While clinical trials provide the highest level of evidence, they are costly and time‐intensive [46, 47]. In contrast, advanced in vitro models allow controlled simulation of the oro‐facial environment [48, 49]. Fatigue testing and accelerated aging in moist environments, which are known to significantly compromise ceramic strength, can provide predictive insights into long‐term material performance [50, 51, 52, 53]. Such fatigue data serve as a valuable baseline for long‐term clinical performance, and cyclic thermomechanical loading has been validated to reproduce clinically relevant failure modes [48, 54, 55].

Therefore, the present study aimed to evaluate the fatigue performance and failure load of composition ‐gradient multilayer zirconia restorations in molar applications. A standardized preparation and loading protocol with two restoration types—partial‐coverage (PCR) and crown (C)—was investigated at three ceramic thicknesses (0.5 mm, 1.0 mm, and 1.5 mm). The research hypothesis was that (i) restoration type and (ii) ceramic thickness would not significantly affect the failure loads under simulated masticatory conditions.

## 2 Material and Methods

**FIGURE 1:**
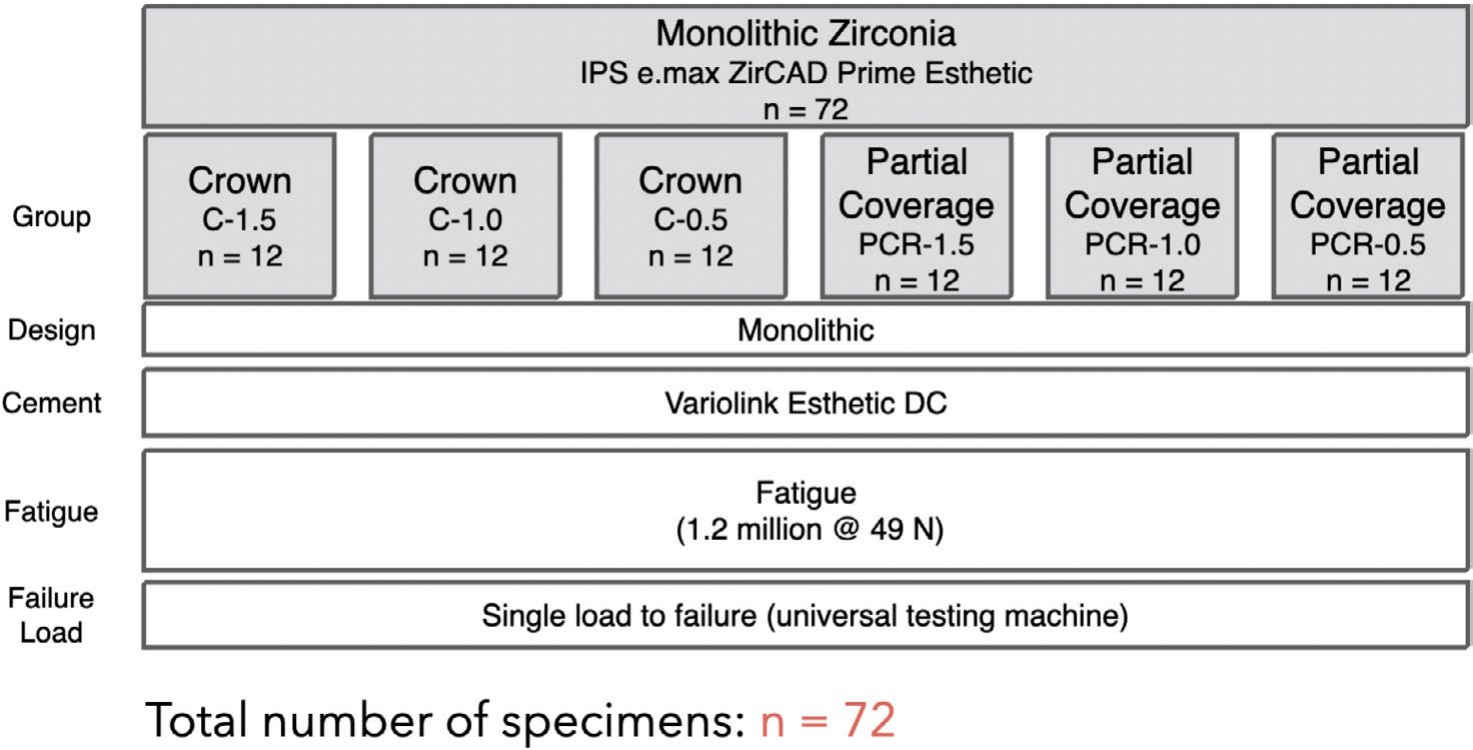
Test‐set up.

In this in vitro study, 72 specimens were randomly assigned to two restoration types: partial‐coverage (PCR, test) and full‐crown (C, control). Each group was further stratified by ceramic thickness (occlusal/buccal: 0.5/0.4 mm, 1.0/0.6 mm, 1.5/0.8 mm), resulting in six subgroups (*n* = 12). The experimental setup is illustrated in Figure 1.

### 2.1 Specimen Preparation

To simulate a clinically relevant scenario, a maxillary first molar from a typodont model (Frasaco, Tettnang, Germany) was used as the basis for all preparations. A single experienced prosthodontist performed all preparations under 4.5× magnification, using coarse and fine diamond burs (no. 370314 035, no. 8370314 035, no. 879314 012, and no. 8879314 012, Komet, Lemgo, Germany) with continuous air–water spray cooling.

**FIGURE 2:**
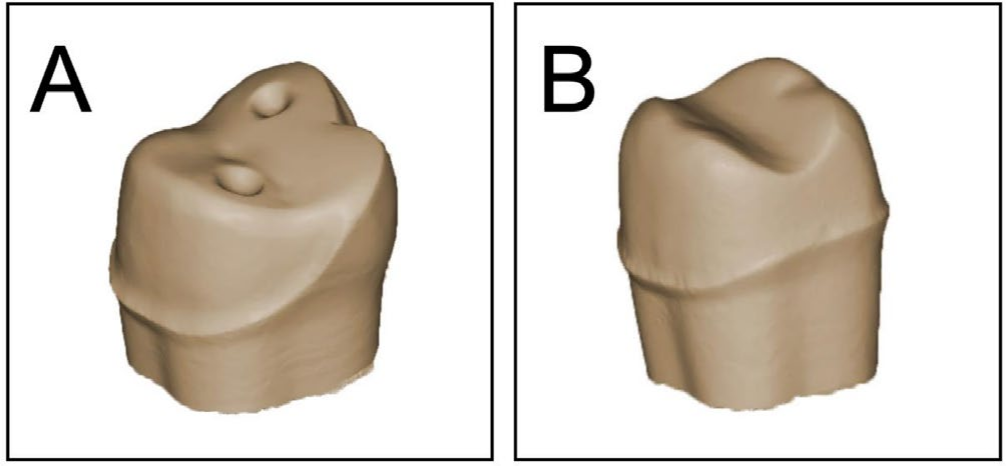
Non‐retentive preparation design of (A) Full‐veneer/Partial‐coverage and (B) Crown.

Six master dies were prepared to represent the two restoration types—partial‐coverage restorations (PCR) and full‐coverage crowns (C)—at three occlusal/buccal ceramic thicknesses: 0.5/0.4 mm, 1.0/0.6 mm, and 1.5/0.8 mm. The PCR design followed a non‐retentive approach, involving occlusal, buccal, and proximal surfaces without box preparations (“full‐veneer design”) [10, 11]. To aid seating and cementation, two shallow diagonal notches (0.2 mm depth) were incorporated into each PCR preparation. A chamfer finish line at the cervical margin was applied in the respective buccal thickness (Figure 2). Crown preparations followed the same dimensional criteria but omitted notches. Preparation depth was verified using silicone keys (TwinDuo, Picodent, Wipperfürth, Germany) and a periodontal probe.

To replicate dentin‐like properties, impressions of the master dies were taken using a polyvinylsiloxane material (Identium, Kettenbach, Eschenburg, Germany), and 72 abutment dies were fabricated from a resin‐based composite (Filtek Z100, 3 M ESPE, Neuss, Germany) with an elastic modulus (~18 GPa) closely matching that of natural dentin [56, 57]. The material was applied in 1.5 mm layers and cured for 20 s per layer using an LED light‐curing unit (Bluephase G4, Ivoclar Vivadent, Schaan, Liechtenstein; 1200 mW/cm [2]).

Following fabrication, the resin dies were stored in distilled water at 37°C for 3 to 5 weeks to promote hydration and ongoing polymerization [58]. Finally, each specimen was embedded in a self‐curing epoxy resin (RenCastCW20 / Ren HY 49, Huntsman, TX, USA) to simulate the elastic support of alveolar bone [59, 60].

### 2.2 Fabrication of All‐Ceramic CAD/CAM Restorations

The six master dies were scanned in occlusion using a lab scanner (PrograScan PS5, Ivoclar Vivadent). Restorations were digitally designed at their designated thicknesses in CAD software (DentalCAD, exocad, Darmstadt, Germany), with a virtual spacer of 50 μm. All restorations were fabricated from a composition of multilayer zirconia (IPS e.max ZirCAD Prime Esthetic, Ivoclar Vivadent), which features a seamless gradient structure composed of 4Y‐PSZ in the dentin zone and 5Y‐PSZ in the incisal zone. The incisal zone measures 3 mm, followed by a 4 mm transition zone (no discrete layers), with the remainder consisting of the dentin zone [61].

**FIGURE 3:**
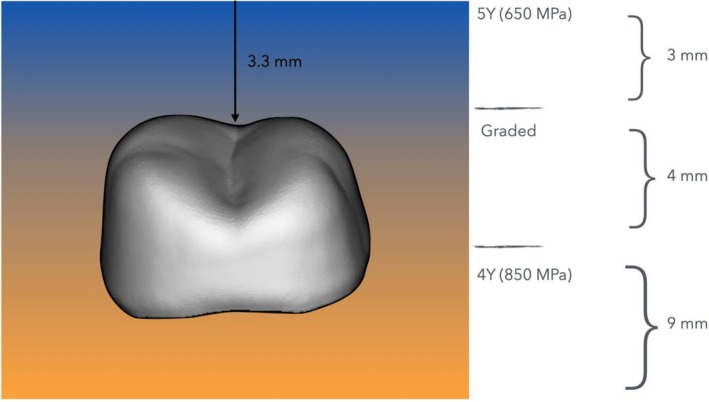
Crowns of each thickness (0.5 mm, 1.0 mm, and 1.5 mm) were placed within the transition layer of the gradient multilayered zirconia blank, maintaining a distance of 3.3 mm from the upper border of the blank to the top surface of the crown.

**FIGURE 4:**
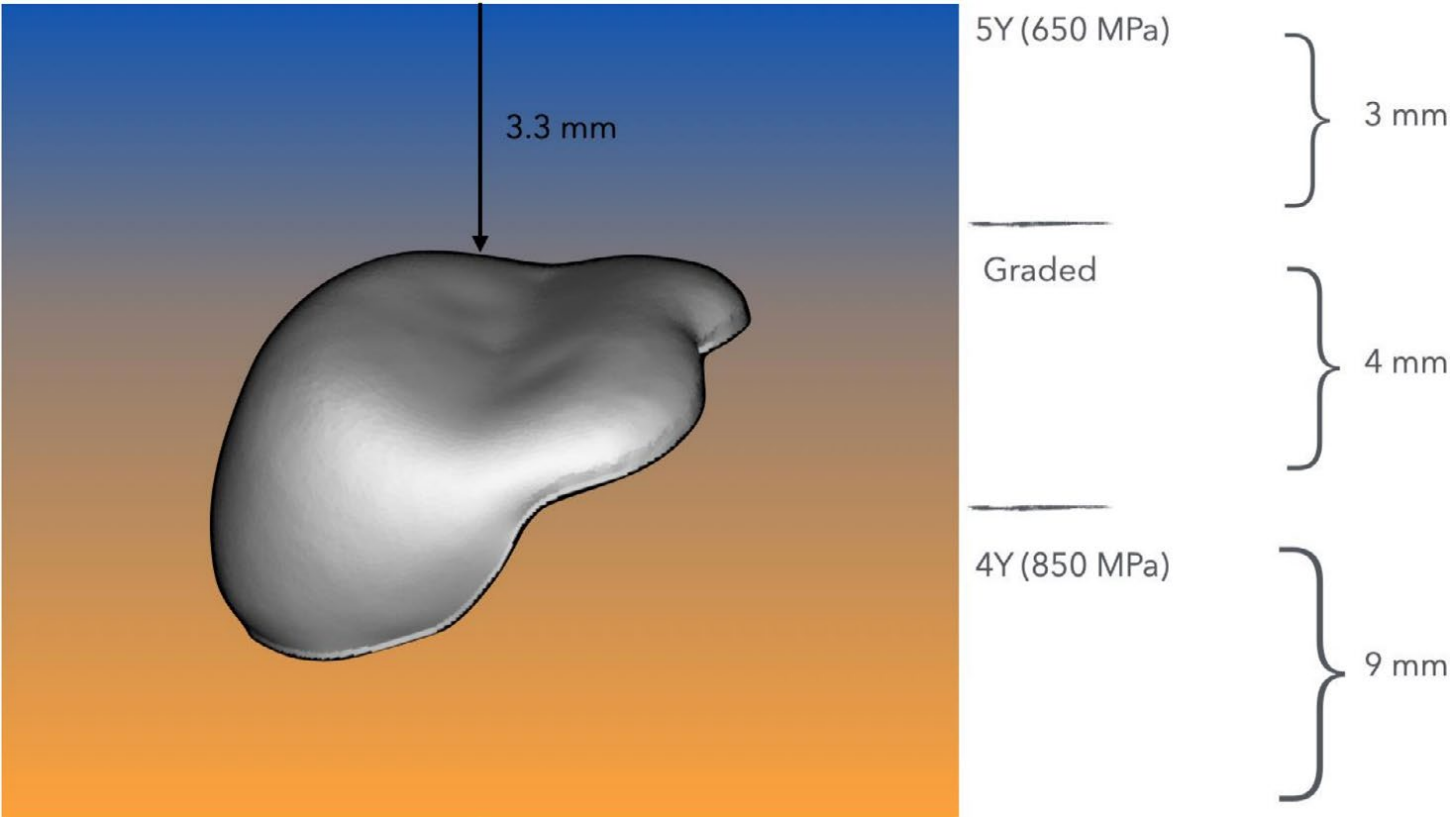
Full‐veneers/Partial‐coverage restorations of each thickness (0.5 mm, 1.0 mm, and 1.5 mm) were placed within the transition layer of the gradient multilayered zirconia blank, maintaining a distance of 3.3 mm from the upper border of the blank to the top surface of the Full‐veneer/Partial‐coverage restoration.

The occlusal surface of each restoration was positioned within the transition layer, 3.3 mm from the top surface of the blank (Figures 3 and 4). Designs were finalized using CAM software (PrograMill CAM, Ivoclar Vivadent) and milled from pre‐sintered multilayered zirconia blanks (IPS e.max ZirCAD Prime Esthetic, Shade A2) using a five‐axis machine (PrograMill PM7, Ivoclar Vivadent). All restorations were fabricated by a single master dental technician in accordance with the manufacturer's guidelines, and layer thickness was verified using a precision caliper (Kroeplin GmbH, Schlüchtern, Germany).

### 2.3 Adhesive Cementation

Adhesive cementation was performed in strict accordance with the manufacturer's instructions [61]. The intaglio surfaces of all gradient multilayered zirconia restorations were air‐particle abraded using 50 μm aluminum oxide at 1 bar for 10 s, followed by the application of a phosphate monomer‐containing ceramic primer (Monobond Plus, Ivoclar Vivadent) for 60 s [62]. Resin dies were pretreated with pumice (Picodent, Wipperfürth, Germany), rinsed with air‐water spray, dried with oil‐free air, and cleaned with 70% ethanol. A light‐curing adhesive (Adhese Universal, Ivoclar Vivadent) was applied to the dies for 20 s, air‐dried, and light‐cured for another 20 s (Bluephase G4, Ivoclar Vivadent; 1200 mW/cm [2]). Adhesive cementation was performed using a dual‐cure resin cement (Variolink Esthetic DC, Ivoclar Vivadent). Proper positioning was guided for PCR restorations by the diagonal occlusal notches. After excess cement removal, margins were covered with glycerin gel (Liquid Strip, Ivoclar Vivadent) and light‐cured for 20 s from each side. To ensure complete polymerization, all specimens were stored in distilled water at 37°C for 24 h (Universalschrank UF55, Memmert) [63].

### 2.4 Fatigue Analysis

All specimens were subjected to cyclic mechanical loading combined with thermocycling (5°C–55°C in distilled water, 120 s dwell time) using a chewing simulator (CS 4.8, SD Mechatronik, Feldkirchen‐Westerham, Germany). To simulate natural mastication, a 49 N occlusal load was applied to the disto‐palatal cusp for 1.2 million cycles at 1.6 Hz, with steatite spheres (*r* = 3 mm; Hoechst CeramTec, Wunsiedel, Germany) moving 0.5 mm horizontally from the cusp to the central fissure [64]. This in vitro setup mimics an aging process equivalent to five years of clinical use under artificial conditions [50, 65, 66, 67]. A transilluminating detection probe (DIA Stick, I.C. Lechner GmbH & Co KG, Stockach, Germany) was used to inspect the specimens at least twice daily for cracks, fractures, or debonding. Survival and success rates were calculated based on damage: specimens with no damage were considered as successes, those with cracks or debonding but still functional were rated as survivals, and those with catastrophic fractures were classified as non‐survival [11, 68, 69].

### 2.5 Single‐Load‐To‐Failure Testing

Following fatigue, all specimens underwent single‐cycle load‐to‐failure (SLF) testing using a universal testing machine (Zwick Z010/TN2S, Zwick Roell, Ulm, Germany). A steel ball (*r* = 3 mm) applied axial load at the same contact point used during fatigue, with a crosshead speed of 1.5 mm/min. A video camera monitored failure progression. Failure was defined as a visible crack, fracture, or a ≥ 20% drop in maximum load (Fmax) without a visible event. The maximum load at failure was recorded using the system's software (TestXpert III, Zwick Roell).

### 2.6 Failure and Fractographic Analysis

**FIGURE 5:**
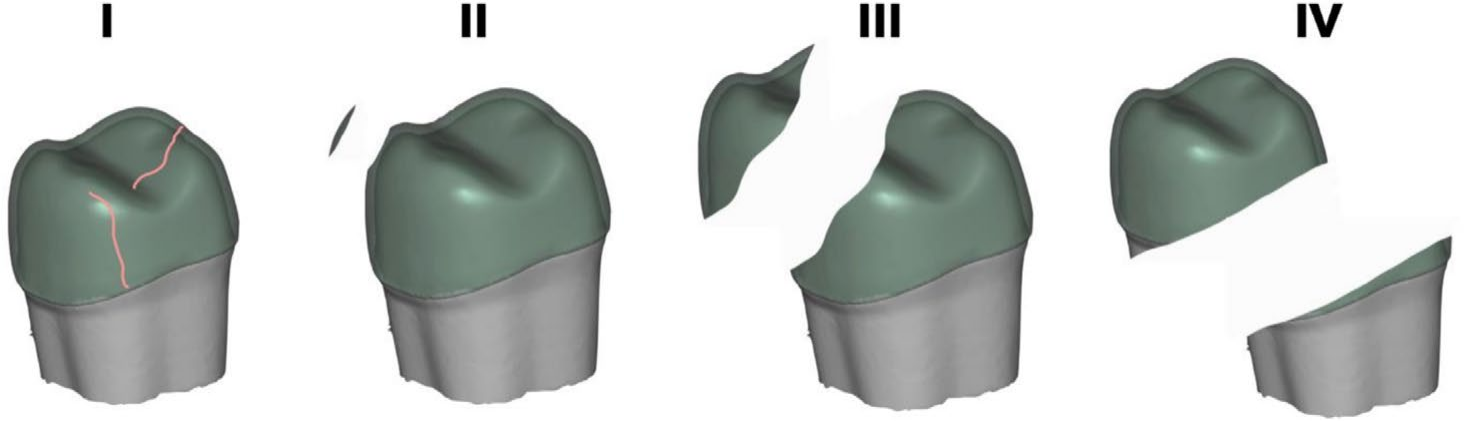
Failure modes after single load to failure testing: (I) Crack formation within the ceramic. (II) Cohesive fracture within the ceramic, intact tooth. (III) Fracture within ceramic and tooth structure. (IV) Serious/longitudinal tooth fracture involving the root.

Failure mode and fracture origin were first assessed using a polarized light microscope (AxioZoom V.16, Zeiss, Oberkochen, Germany). Z‐stack imaging (ZEN Core 3.3, Zeiss) was used to enhance depth of field by merging images at different focal planes. Representative fracture patterns were further examined via qualitative fractographic analysis using scanning electron microscopy (Vega 3, Tescan, Kohoutovice, Czech Republic). Failure modes were categorized as follows: (I) Crack formation within the ceramic; (II) cohesive ceramic fracture with an intact tooth; (III) combined fracture of ceramic and tooth structure; and (IV) severe longitudinal tooth fracture extending into the root (Figure 5).

### 2.7 Statistical Analysis

A power analysis (G*Power 3.1.9.7, Düsseldorf, Germany) for a 2 × 3 factorial design—restoration type (PCR vs. crown) and ceramic thickness (0.5, 1.0, 1.5 mm)—determined a sample size of *n* = 12 per group (*n* = 72 total) to detect medium effect sizes (f = 0.26) with 80% power and α = 0.05. Statistical analysis was performed using SPSS 28 (IBM, Armonk, NY, USA). Levene's test confirmed the homogeneity of variances. ANOVA was used to assess main effects and interactions, followed by Tukey post hoc tests (per design) and two‐sample t‐tests (per thickness) where appropriate. Significance was set at *p* < 0.05 (95% CI). Results were visualized using boxplots.

## 3 Results

### 3.1 Fatigue Performance

**FIGURE 6:**
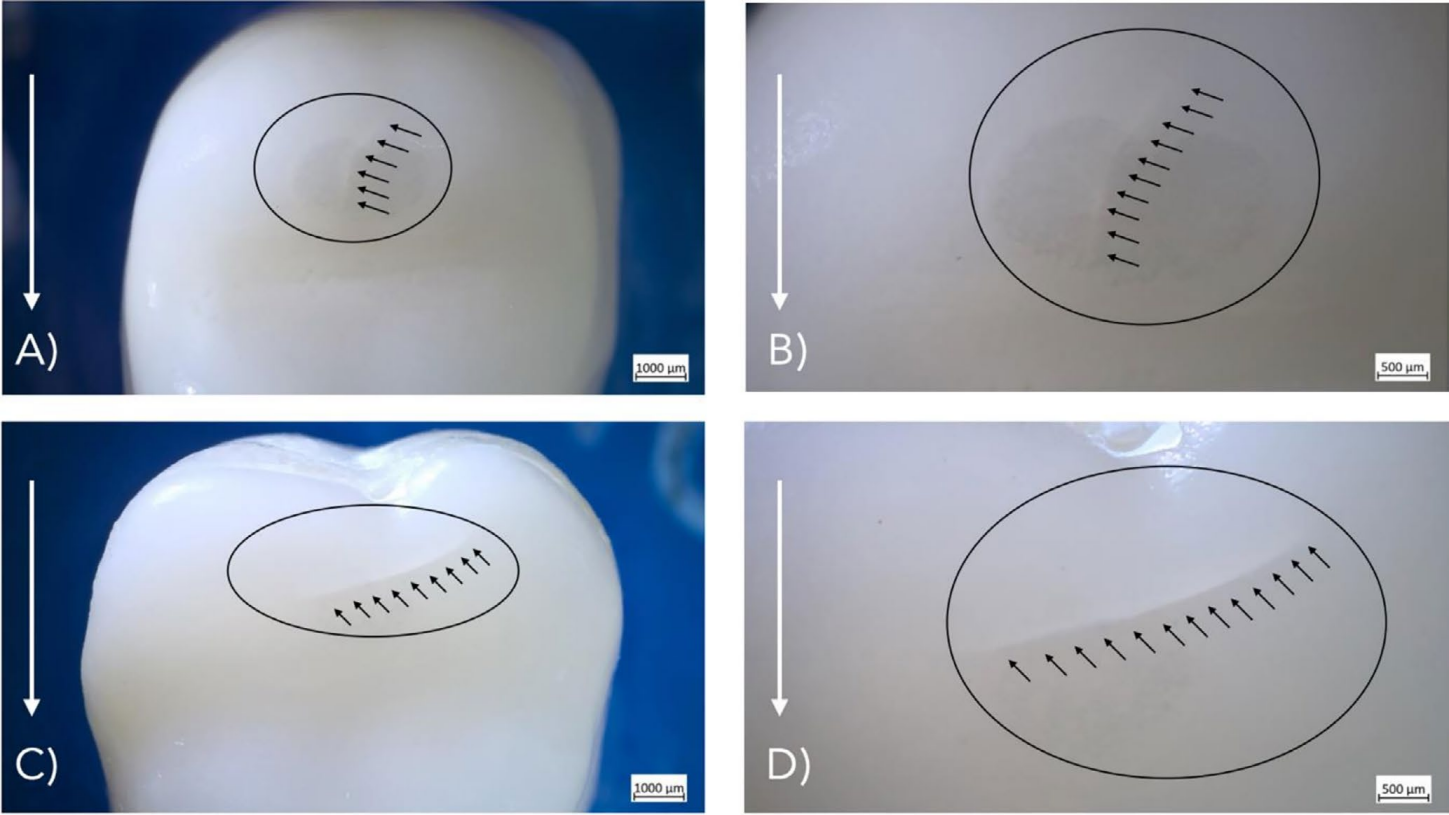
Light microscope images of 0.5 mm thick gradient multilayered zirconia restorations showing cracks (small arrows) and wear facets (circle) after fatigue exposure. Bold arrow indicates direction of sliding movement during fatigue. (A and B) C‐0.5 Sample No. 10: One crack after 802,485 cycles; (C and D) PCR‐0.5 Sample No. 10: One crack after 551,960 cycles.

All restorations exhibited wear facets at the contact area due to steatite ball movement during cyclic loading. Both restoration types showed a 100% survival rate, with no debonding or catastrophic fractures observed during or after fatigue. However, not all specimens remained free of damage: one crown of Group C‐0.5 and one restoration in Group PCR‐0.5 developed visible cracks during fatigue (Figure 6), resulting in an overall success rate of 97.22%. Detailed success rates by ceramic thickness are presented in Table 1.

**TABLE 1:** Success rates after simulated fatigue exposure of 5 years.

Group	Intact and unharmed specimens after fatigue	Success rate	Success rate of restoration design	Overall success rate
C‐1.5	12/12	100%	Crowns	97.22%
C‐1.0	12/12	100%	97.22%	
C‐0.5	11/12 (one crack after 802,485 cycles)	91.67%	35/36	
PCR‐1.5	12/12	100%	PCRs	
PCR‐1.0	12/12	100%	97.22%	
PCR‐0.5	11/12 (one crack after 551,960 cycles)	91.67%	35/36	

### 3.2 Single Load to Failure

**FIGURE 7:**
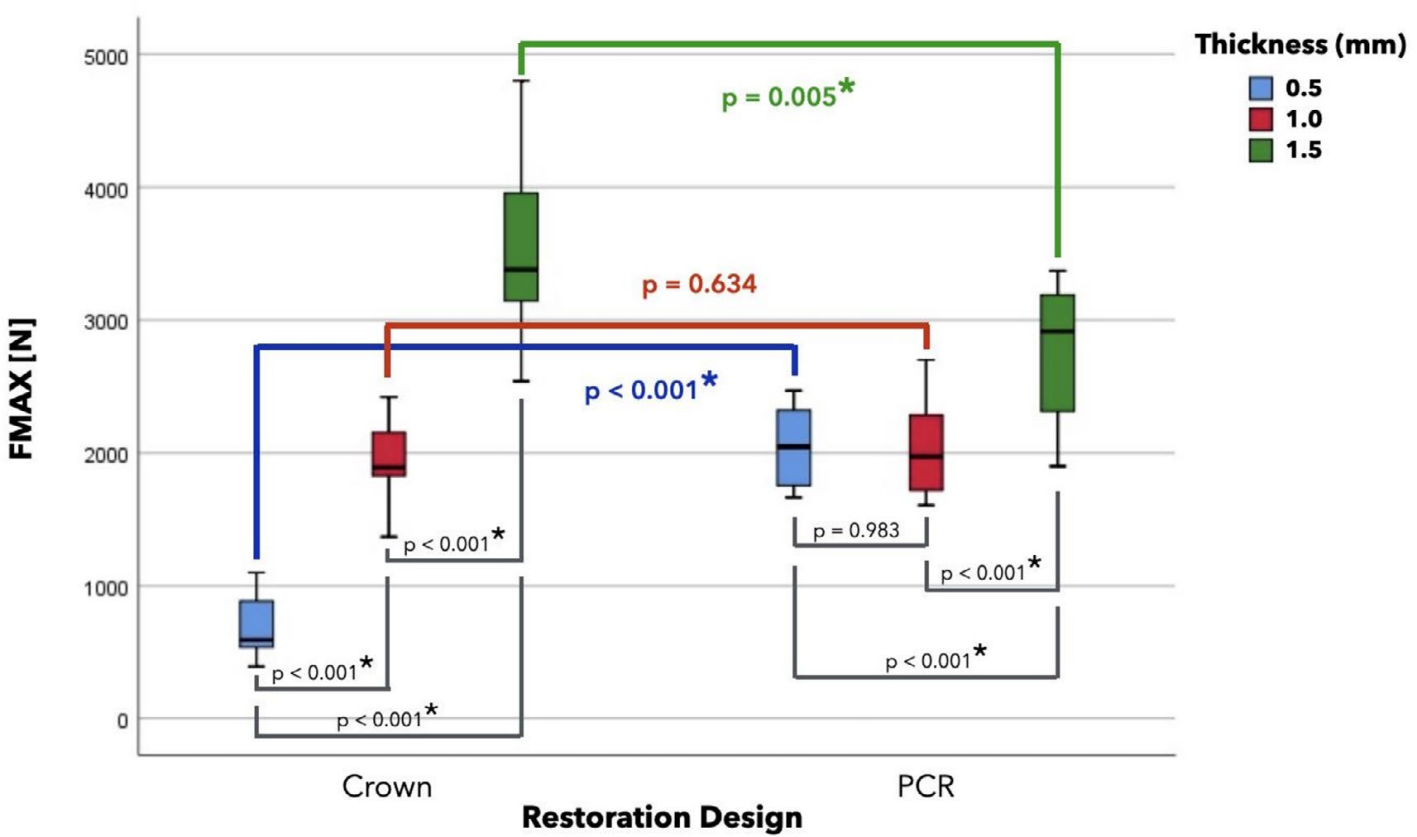
Boxplot of failure loads (F_max_ in N) of tested groups. Statistical significance (*p* < 0.05) is indicated by an asterisk in front of the *p‐*value.

Failure load values after SLF are summarized in Table 2 and illustrated in Figure 7. The mean failure loads (N) are ranked as follows: C‐1.5 (3503) > PCR‐1.5 (2777) > PCR‐0.5 (2047) > PCR‐1.0 (2018) > C‐1.0 (1957) > C‐0.5 (695).

**TABLE 2:** Failure load results of all tested groups [*N* = Newton]. Min = minimum; 1st Qu = 25% of data was below this value; Median = 50% of data was below this value; 3rd Qu = 75% of data was below this value; Max = maximum; SD = Standard deviation.

Group name	Min	1st Qu	Median	Mean	3rd Qu	Max	SD
C‐1.5	2540	3104	3380	3503	3902	4800	628
C‐1.0	1370	1783	1890	1957	2130	2420	274
C‐0.5	393	553	594	695	837	1101	224
PCR‐1.5	1900	2453	2915	2777	3100	3370	509
PCR‐1.0	1606	1798	1974	2018	2238	2700	346
PCR‐0.5	1665	1857	2047	2047	2236	2470	298

Both restoration type [F (1, 66) = 5.72, *p* = 0.02, partial eta^2^ = 0.80] and ceramic layer thickness [F (1, 66) = 117.63, *p* < 0.001, partial eta^2^ = 0.78] had a statistically significant effect on failure load. These main effects were qualified by a crossed interaction between restoration type and ceramic layer thickness [F (2, 66) = 40.14, *p* < 0.001, partial eta^2^ = 0.55], reflecting the fact that the relative failure load advantage for crowns vs. PCRs at the 1.5 mm thickness level was reversed into a relative disadvantage at 0.5 mm.

Post hoc Tukey tests, computed per restoration design, showed that increasing ceramic thickness strongly improved failure load in crowns (C‐0.5 vs. C‐1.0: *p* < 0.001; C‐0.5 vs. C‐1.5: *p* < 0.001; C‐1.0 vs. C‐1.5: *p* < 0.001). In PCRs, the thickest variants also demonstrated significantly higher failure loads than did thinner variants (PCR‐0.5 vs. PCR‐1.5: *p* < 0.001; PCR‐1.0 vs. PCR‐1.5: *p* < 0.001), whereas no difference was observed between 0.5 mm and 1.0 mm thicknesses (*p* = 0.983). Post hoc t‐tests comparing restoration designs at each ceramic thickness level revealed that ultrathin PCRs (0.5 mm) had significantly higher failure loads than ultrathin crowns (*t* = −12.55, *p* < 0.001), while at 1.5 mm thickness, crowns outperformed PCRs (*t* = 3.12, *p* = 0.005). No significant difference between restoration designs was found at 1.0 mm thickness (t = −0.48, *p* = 0.63).

### 3.3 Failure and Fractographic Analysis

Fatigue failure analysis identified radial cracks in one specimen each from Group C‐0.5 and Group PCR‐0.5, originating from the intaglio cementation surface beneath the contact point. The cracks were confined to the ceramic layer and did not reach the occlusal surface (Figure 7).

**FIGURE 8:**
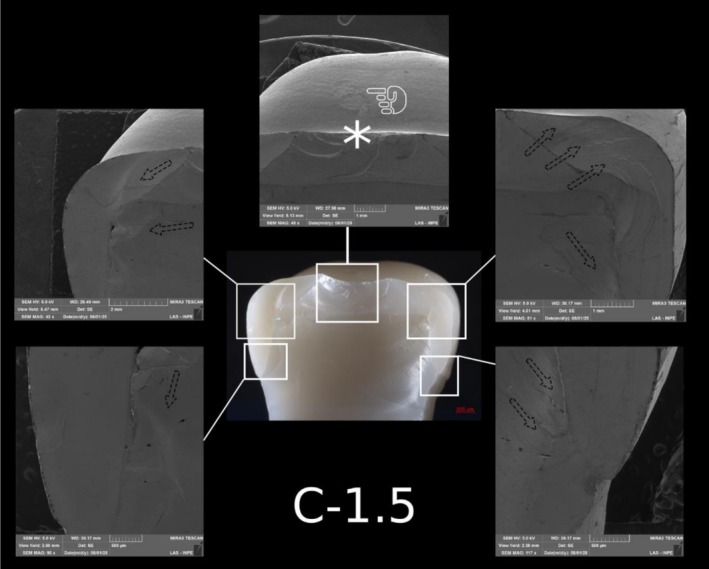
Representative specimen of the C‐1.5 group after the single‐load‐to‐fracture test. Light microscopy shows a buccal overview of the bulk‐fractured specimen, while SEM images at different magnifications demonstrate fractography marks and the wear scar (pointer). The asterisk indicates the possible fracture origin site, and the dotted arrows indicate the direction of crack propagation.

**FIGURE 9:**
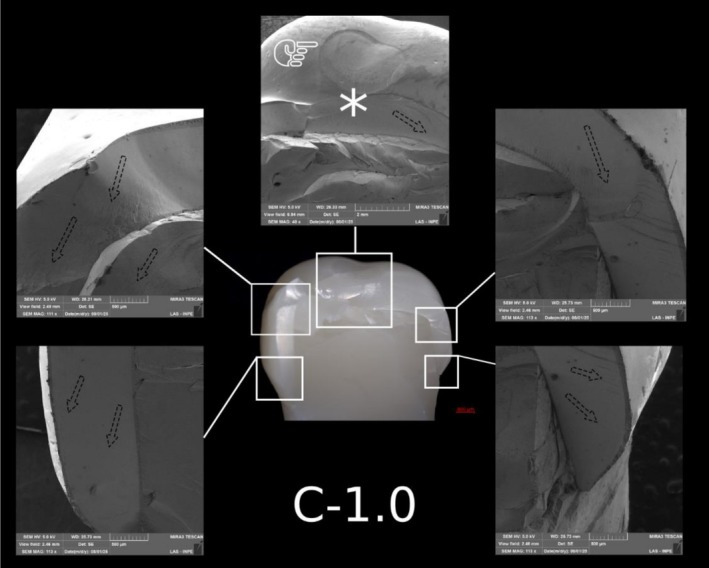
Representative specimen of the C‐1.0 group after the single‐load‐to‐fracture test. Light microscopy shows a buccal overview of the bulk‐fractured specimen, while SEM images at different magnifications demonstrate fractography marks and the wear scar (pointer). The asterisk indicates the possible fracture origin site, and the dotted arrows indicate the direction of crack propagation.

**FIGURE 10:**
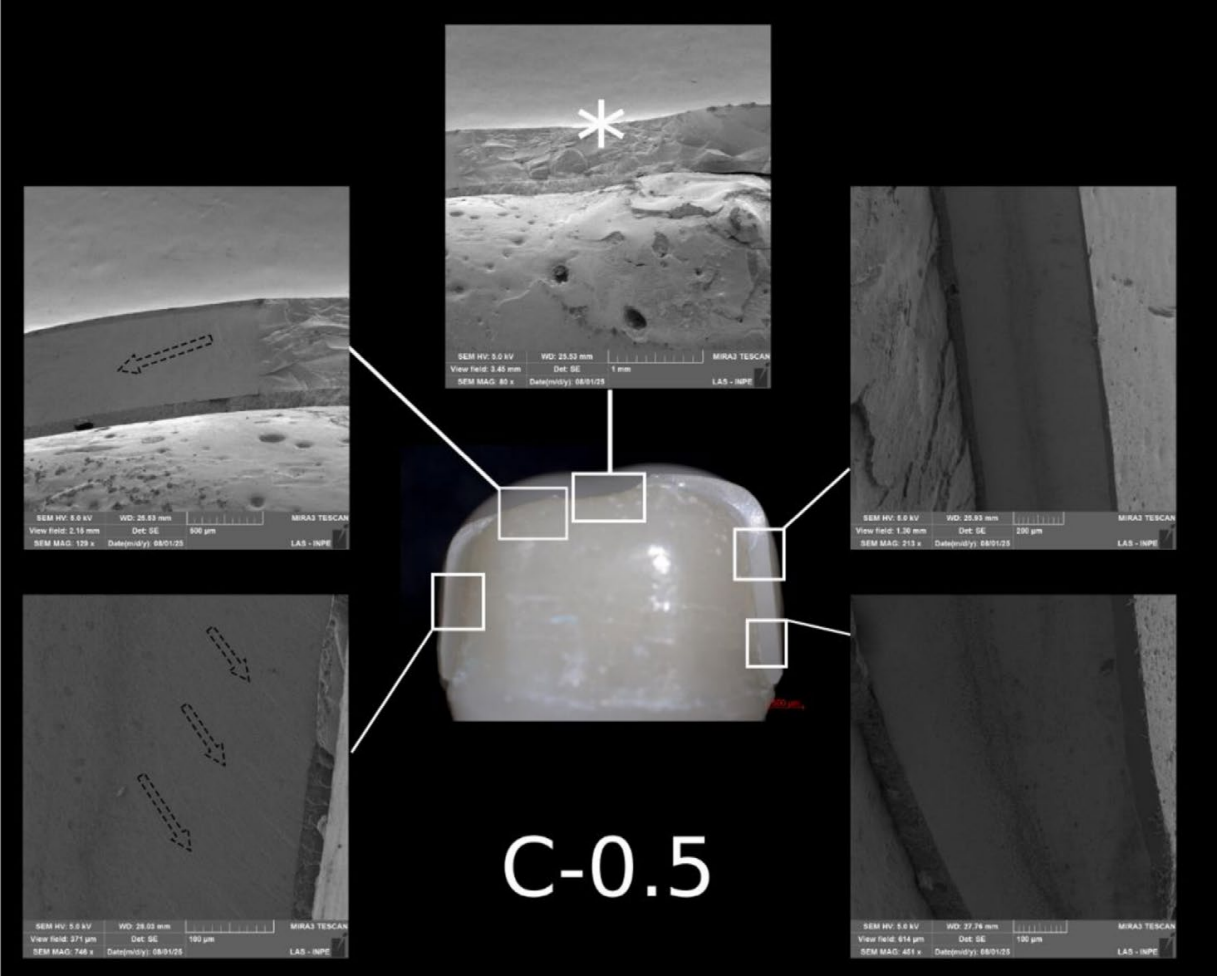
Representative specimen of the C‐0.5 group after the single‐load‐to‐fracture test. Light microscopy shows a buccal overview of the bulk‐fractured specimen, while SEM images at different magnifications demonstrate fractography marks. The asterisk indicates the possible fracture origin site, and the dotted arrows indicate the direction of crack propagation.

**FIGURE 11:**
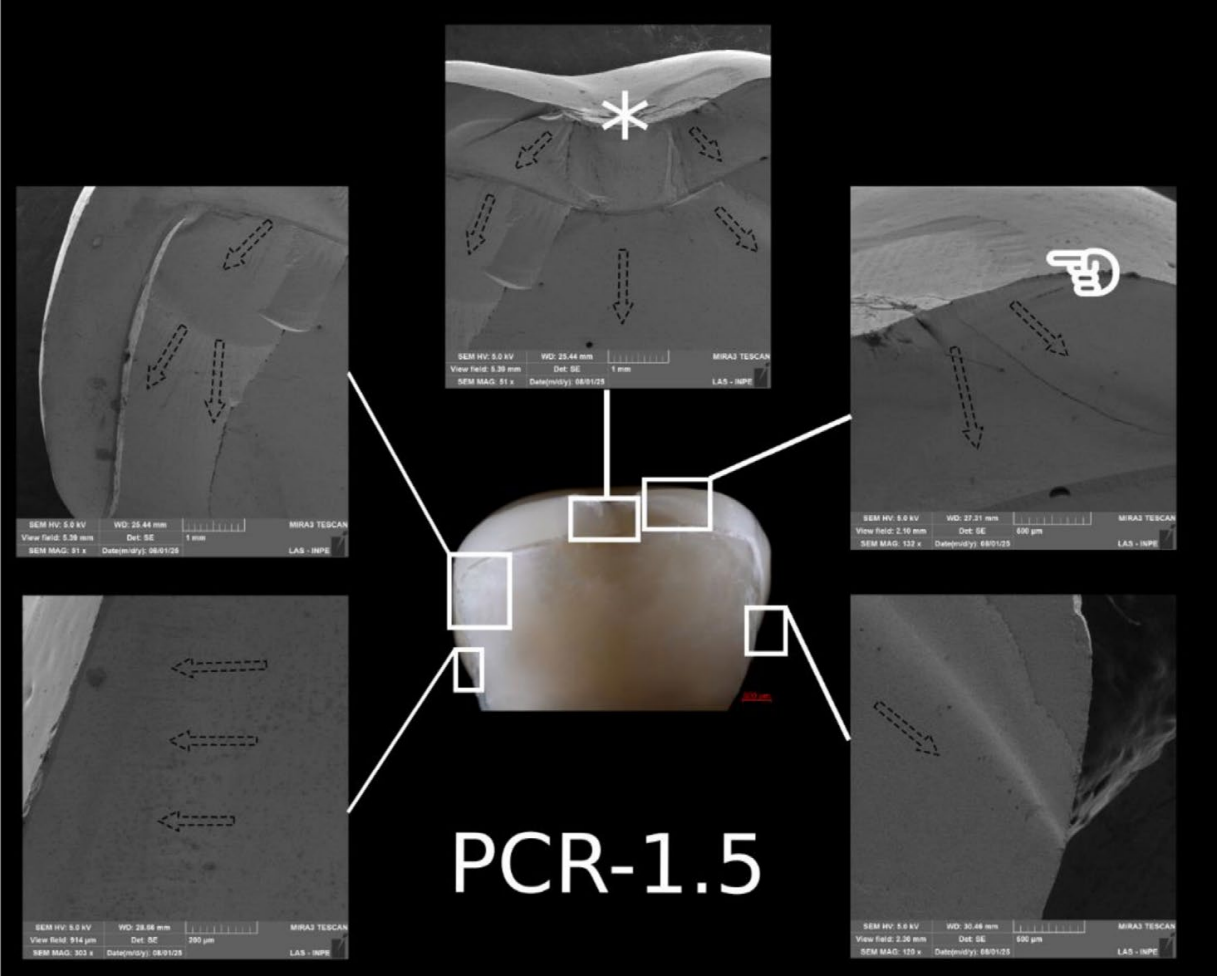
Representative specimen of the PCR‐1.5 group after the single‐load‐to‐fracture test. Light microscopy shows a buccal overview of the bulk‐fractured specimen, while SEM images at different magnifications demonstrate fractography marks and the wear scar (pointer). The asterisk indicates the possible fracture origin site, and the dotted arrows indicate the direction of crack propagation.

**FIGURE 12:**
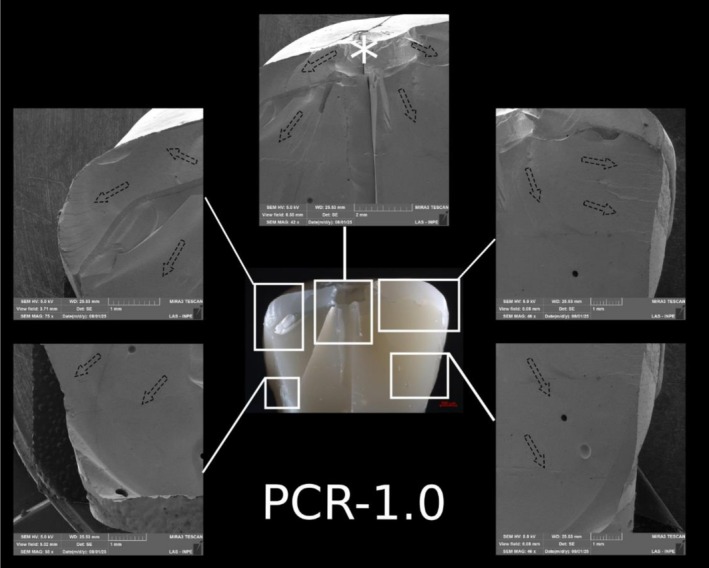
Representative specimen of the PCR‐1.0 group after the single‐load‐to‐fracture test. Light microscopy shows a buccal overview of the bulk‐fractured specimen, while SEM images at different magnifications demonstrate fractography marks. The asterisk indicates the possible fracture origin site, and the dotted arrows indicate the direction of crack propagation.

**FIGURE 13:**
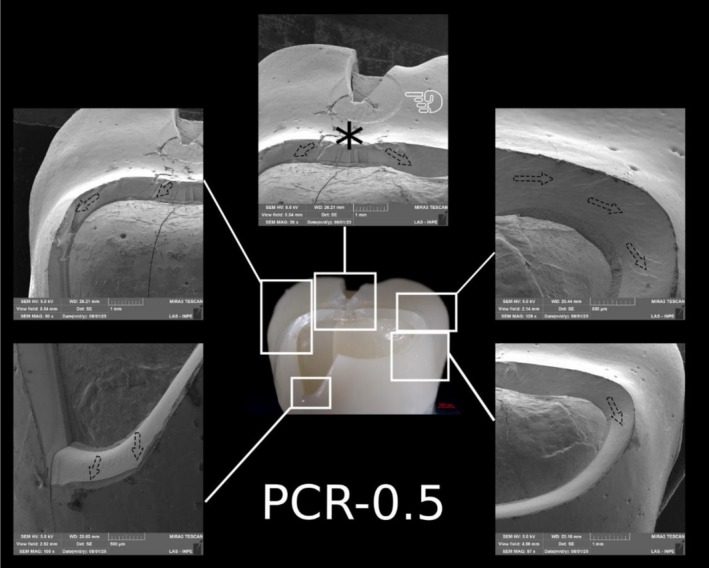
Representative specimen of the PCR‐0.5 group after the single‐load‐to‐fracture test. Light microscopy shows a buccal overview of the bulk‐fractured specimen, while SEM images at different magnifications demonstrate fractography marks and the wear scar (pointer). The asterisk indicates the possible fracture origin site, and the dotted arrows indicate the direction of crack propagation.

Post‐SFL analysis revealed two distinct fracture modes: crowns in Groups C‐1.5 and C‐1.0 predominantly exhibited severe longitudinal fractures with involvement of the resin die (Type IV), while ultrathin crowns in Group C‐0.5 mainly showed cohesive ceramic fractures with intact dies (Type II). In contrast, PCR specimens, regardless of thickness, primarily demonstrated combined ceramic and die fractures (Type III) (Table 3). Detailed fractography analysis of representative specimens from each group is presented in Figures 8, 9, 10, 11, 12, 13. Light microscopy provides a buccal overview of the bulk‐fractured specimens, while SEM micrographs reveal fractography features, including hackle lines, indicating the possible fracture origin and the crack propagation.

**TABLE 3:** Occurrence of failure modes for each group in %. Failure modes were classified as follows: (I) crack formation within the ceramic, (II) cohesive fracture within the ceramic, intact tooth, (III) fracture within ceramic and tooth structure, (IV) serious/longitudinal tooth fracture involving the root.

Group name	Failure mode (%)			
	I	II	III	IV
C‐1.5	0	0	25.0	75.0
C‐1.0	0	0	33.3	66.7
C‐0.5	0	83.4	16.7	0
PCR‐1.5	0	8.3	75.0	16.7
PCR‐1.0	0	0	91.7	8.3
PCR‐0.5	0	16.7	83.3	0

## 4 Discussion

This in vitro study investigated the fatigue performance of composition strength‐gradient multilayer zirconia restorations under simulated masticatory loading, with a focus on restoration type (PCR vs. crown) and ceramic thickness (occlusal/buccal 0.5/0.4 mm, 1.0/0.6 mm, 1.5/0.8 mm). The results provide valuable insights into the applicability of ultrathin zirconia ceramics in minimally invasive prosthodontic procedures.

The research hypothesis was rejected, as both restoration type and ceramic thickness significantly influenced failure load. All specimens survived fatigue testing (100% survival rate), with a high overall success rate (97.2%). Only two specimens developed visible cracks during fatigue—one from group C‐0.5 (crack at 802,485 cycles) and one from group PCR‐0.5 (crack at 551,960 cycles). These results align with recent clinical studies reporting high survival rates (99.1%–100%) for monolithic zirconia single crowns in posterior regions after 3–7 years of service [16, 18]. Early clinical evidence indicates that zirconia veneers in the anterior dentition demonstrate excellent performance, with 100% survival after up to three years [17, 19]. However, so far no clinical data are available for monolithic zirconia PCRs.

With the exception of Group C‐0.5, all tested specimens demonstrated failure loads exceeding the normal masticatory force range in the posterior region (289–700 N), indicating adequate mechanical strength for clinical application in high‐load areas such as molars [46]. Although 0.5 mm crowns showed high survival and success after aging, their lower failure load (~695 N) nears the upper threshold of physiological and parafunctional forces (up to 900 N), suggesting potential limitations under intensified load conditions [47].

Zirconia restorations are known for their high flexural strength and fracture toughness [15]; however, **restoration thickness is a critical factor** influencing their load‐bearing capacity [27]. Thinner restorations provide less bulk to resist crack propagation, making them more susceptible to mechanical failure under high occlusal loads [55, 67]. Studies have shown that mechanical strength decreases non‐linearly for monolithic zirconia crowns with reduced ceramic thickness, especially below 1 mm [27, 67]. This might be attributed to higher tensile stresses at the intaglio surface and potential flaws introduced during manufacturing, cementation, or intraoral function (repetitive loading and damage accumulation), which become more critical as material thickness decreases [15, 55, 67].

Load‐to‐failure testing confirmed that both restoration type and ceramic thickness significantly affected failure loads. As anticipated, increasing ceramic thickness improved load‐bearing capacity in both PCRs and crowns, consistent with prior findings that demonstrate a direct correlation between material thickness and mechanical strength [11, 67]. Notably, at the lowest thickness level (0.5 mm), PCRs significantly outperformed crowns (2047 N vs. 695 N, *p* < 0.001), suggesting a mechanical advantage of the non‐retentive PCR design under ultrathin conditions. This may result from more favorable stress distribution and lower internal tension during loading, as restoration design complexity and tooth structure loss critically affect the integrity of the tooth–restoration system [3].

At a thickness of 1.0 mm, no significant differences were observed between the two restoration types, indicating comparable fatigue performance in thin‐thickness applications. In contrast, crowns exhibited superior fracture resistance at 1.5 mm thickness (*p* = 0.005), suggesting that increased thickness enhances the crown's ability to withstand compressive and tensile forces [67]. These findings underscore the complex interaction between design geometry, ceramic thickness, and load transmission pathways in determining structural performance [52]. Therefore, PCRs seem to be clinically suitable at all tested thicknesses, whereas crowns should have a minimum thickness of 1 mm when applied in molar restorations.

Comparative in vitro studies have yielded complementary findings for different PCR designs, variation of yttria content of zirconia and ceramic thickness for crowns. In one investigation, various 3Y‐TZP (ZirCAD LT) PCR designs with 1 mm occlusal thickness were applied to human premolars and subjected to thermomechanical loading (1.2 million cycles, 70 N, 1.4 Hz), with crowns serving as controls [38]. Despite adherence to the recommended APC bonding protocol (air‐particle abrasion, primer application, composite cement) for zirconia restorations, one PCR involving occlusal, proximal, and buccal coverage debonded during testing [62]. However, this study did not include failure load assessment after fatigue. Resin bonding has been shown to benefit zirconia restorations, especially in cases with reduced thickness, low mechanical retention, or high shear forces [23]. Long‐term success of zirconia restorations relies on proper surface pretreatment, including air‐particle abrasion, MDP‐based (10‐methacryloyloxydecyl dihydrogen phosphate) primers, and suitable self‐ or dual‐cure composite cements [23].

Recent investigations on minimally invasive monolithic occlusal veneers fabricated from composition strength‐gradient multilayer zirconia with thicknesses ranging from 0.3 to 0.6 mm (ZirCAD Prime) [43] and 0.5–1.5 mm (ZirCAD Prime Esthetic) [45] demonstrated that all tested specimens exhibited post‐fatigue failure loads (1.2 million cycles, 49 N at 1.6 Hz [45] and 98 N at 2.4 Hz [43]) exceeding both physiological and parafunctional masticatory forces (≤ 900 N). Nevertheless, veneers made entirely of 5Y‐PSZ showed isolated failures (one specimen) and partial failures in 7.8% of specimens after fatigue, whereas 3Y‐TZP, 4Y‐PSZ, and 3Y‐TZP + 5Y‐PSZ groups remained intact, independent of whether bonding occurred to enamel or dentin [43]. The success rate of the 0.5 mm occlusal veneers decreased to 50% compared with the 1.0 mm and 1.5 mm groups, as half of the specimens developed cracks or surface damage after cyclic loading, indicating that thinner restorations require particular caution due to their reduced fatigue resistance [45].

Another study tested full‐contour molar crowns made from 3Y‐TZP (ZirCAD LT), 4Y‐PSZ (ZirCAD MT), and strength‐gradient multilayer zirconia (5Y‐PSZ/3Y‐TZP) (ZirCAD Prime) and 5Y‐PSZ/4Y‐PSZ (ZirCAD MT Multi) with 1.5 mm occlusal thickness following artificial aging (1.2 million cycles, 70 N, 1.4 Hz) [34]. No fatigue‐induced failures were observed confirming the findings of the present study for 1.5 mm crowns. Failure load values were highest for 3Y‐TZP (3708 N) and 4Y‐PSZ (3172 N), followed by the multilayer variants (5Y‐PSZ/3Y‐TZP: 2461 N; 5Y‐PSZ/4Y‐PSZ: 2393 N). The authors concluded that the yttria concentration at the occlusal surface is the primary determinant of fracture resistance, irrespective of the supporting zirconia layer [34]. The higher failure load observed in Group C‐1.5 (3503 N) of the present study compared to the cited study may be attributed to the specific positioning of the restoration within the gradient structure of the multilayer zirconia, which influences its mechanical strength and fatigue resistance [35].

A comparable laboratory investigation evaluated monolithic zirconia molar crowns with a 1 mm occlusal thickness under identical fatigue conditions and observed a clear decline in failure load with increasing yttria content: 3Y‐TZP (7530 N, DD Bio ZX2) > 4Y‐PSZ (5000 N, DD cubeX2 HS) > 5Y‐PSZ (3700 N, DD cubeX2) [22]. This trend reflects the reduction in the tetragonal phase in higher yttria‐content zirconia, which diminishes transformation toughening—a mechanism wherein stress‐induced tetragonal‐to‐monoclinic phase transformation at crack tips produces localized volumetric expansion, generating compressive stresses that impede crack propagation [15, 20, 32]. The notably high failure loads in that study may also be attributed to the use of metal abutments, which provide higher stiffness than dentin or composite resin, thereby reducing strain concentrations during loading [22].

Another in vitro study tested 0.5 mm thick molar crowns made of various zirconia compositions—3Y‐TZP (DD Bio ZX2), 4Y‐PSZ (DD Cube ONE), 4Y‐PSZ multilayer (DD Cube ONE ML), and 5Y‐PSZ (DD cubeX2)—after artificial aging (1.2 million cycles, 50 N) [53]. In contrast to the present study, no failures were observed post‐fatigue, with higher failure loads ranging from 1211 N (5Y‐PSZ) to 3952 N (4Y‐PSZ ML) compared to the tested 0.5 mm crowns, which failed at 695 N.

However, direct comparison to the present findings is limited, as this is the first in vitro study evaluating full‐veneer molar crowns made from composition‐gradient multilayer zirconia. Variability in abutment materials, preparation geometry, loading protocols, and cementation techniques complicate direct benchmarking across studies [44].

While, 4Y‐PSZ exhibits higher flexural strength and fracture toughness due to partial retention of the tetragonal phase and associated transformation‐toughening mechanisms, 5Y‐PSZ achieves greater translucency but reduced mechanical reliability as a result of its predominantly cubic microstructure [15, 20]. Composition strength‐gradient multilayer zirconia combines 4Y and 5Y phases effectively, balancing these opposing properties within a single material, yielding both enhanced optical performance and improved fatigue resistance [32]. This graded architecture may therefore offer superior load‐bearing capacity compared with monolithic 5Y zirconia and superior translucency compared with monolithic 4Y zirconia, providing an optimal compromise between mechanical and esthetic requirements for clinical applications [36].

In addition, resistance to low‐temperature degradation (LTD) remains a key factor influencing zirconia's long‐term clinical performance [31]. It is well established that both 3Y‐TZP and 4Y‐PSZ exhibit susceptibility to LTD due to the presence of transformable tetragonal grains, which undergo t → m transformation [24, 30, 39]. In contrast, 5Y‐PSZ, owing to its predominantly cubic phase content, shows resistance to LTD [28, 29]. Therefore, beyond the established balance between esthetics and strength, incorporating a 5Y top layer in a bilayer or multilayer configuration provides an additional advantage by mitigating aging effects at the restoration surface, thereby contributing to improved long‐term stability and overall restoration survival under intraoral conditions. A bilayer system combining a 4Y‐PSZ core with a 5Y‐PSZ top layer has demonstrated superior mechanical performance, both before and after aging, relative to monolithic 5Y‐PSZ, without jeopardizing translucency [28].

Failure analysis revealed that PCRs mainly exhibited combined ceramic and substrate fractures (Type III), while crowns tended to fail with extensive longitudinal cracks (Type IV). This supports the interpretation that while crowns offer greater structural robustness (at a thickness of 1.0 and 1.5 mm), they may transmit higher stresses to the underlying substrate during catastrophic failure, potentially compromising the remaining tooth structure [11].

Despite the strengths of this study, several limitations must be acknowledged. First, the inherent nature of an in vitro design limits the extrapolation of results to real‐world clinical scenarios. The complex biomechanical and biological interactions in the oral environment cannot be fully replicated under laboratory conditions [49]. Second, resin‐based composite dies, which closely approximate the elastic modulus of natural dentin, were used in place of extracted human teeth to ensure reproducibility and standardization over anatomical variability [11]. Moreover, only one single commercially available composition‐gradient multilayer zirconia system (IPS e.max ZirCAD Prime Esthetic) was tested. While this approach strengthens internal consistency and isolates the performance of one material, it restricts the generalizability of the results to other multilayer zirconia products that may differ in yttria content, gradient architecture, and sintering behavior [15].

In summary, the findings of this study provide meaningful insights into the fatigue performance of composition‐gradient multilayer zirconia restorations under controlled conditions and support the clinical potential for minimally invasive restorations, particularly in posterior regions subjected to high functional loads. Currently, the manufacturer recommends a minimum occlusal thickness of 1.0 mm and restricts its indication to single crowns. However, the superior performance of ultrathin PCRs (0.5 mm) compared to crowns at the same thickness challenges conventional design paradigms and underscores the mechanical viability of conservative restorations when adhesively bonded.

Future research should include a broader range of clinical designs to better inform decision‐making for minimally invasive multilayer zirconia restorations and address clinical trials to validate the in vivo performance and complication rates. Complementary finite element analysis (FEA) may further elucidate stress distribution patterns and optimize restoration design.

## 5 Conclusions

Within the limitations of this current study, it was concluded that: partial‐coverage restorations demonstrated superior failure load after fatigue compared to full‐coverage crowns at 0.5 mm, supporting their use in ultrathin, minimally invasive applications. At 1.0 mm and 1.5 mm, both designs showed reliable performance, with crowns achieving higher failure loads than partial‐coverage restorations at 1.5 mm.

The tested composition‐gradient multilayer zirconia restorations exhibited high fatigue reliability, making them suitable for non‐retentive posterior applications. However, a minimum thickness of 1.0 mm should be maintained for single crowns.

## Funding

This study was funded by a research grant from the International College of Prosthodontists (ICP).

## Conflicts of Interest

F.S. has provided lectures sponsored by Ivoclar Vivadent outside of the submitted work.

## Data Availability

The data that support the findings of this study are available from the corresponding author upon reasonable request.
